# A comprehensive overview of barriers and strategies for AI implementation in healthcare: Mixed-method design

**DOI:** 10.1371/journal.pone.0305949

**Published:** 2024-08-09

**Authors:** Monika Nair, Petra Svedberg, Ingrid Larsson, Jens M. Nygren

**Affiliations:** School of Health and Welfare, Halmstad University, Halmstad, Sweden; Vrije Universiteit Brussel, BELGIUM

## Abstract

Implementation of artificial intelligence systems for healthcare is challenging. Understanding the barriers and implementation strategies can impact their adoption and allows for better anticipation and planning. This study’s objective was to create a detailed inventory of barriers to and strategies for AI implementation in healthcare to support advancements in methods and implementation processes in healthcare. A sequential explanatory mixed method design was used. Firstly, scoping reviews and systematic literature reviews were identified using PubMed. Selected studies included empirical cases of AI implementation and use in clinical practice. As the reviews were deemed insufficient to fulfil the aim of the study, data collection shifted to the primary studies included in those reviews. The primary studies were screened by title and abstract, and thereafter read in full text. Then, data on barriers to and strategies for AI implementation were extracted from the included articles, thematically coded by inductive analysis, and summarized. Subsequently, a direct qualitative content analysis of 69 interviews with healthcare leaders and healthcare professionals confirmed and added results from the literature review. Thirty-eight empirical cases from the six identified scoping and literature reviews met the inclusion and exclusion criteria. Barriers to and strategies for AI implementation were grouped under three phases of implementation (planning, implementing, and sustaining the use) and were categorized into eleven concepts; Leadership, Buy-in, Change management, Engagement, Workflow, Finance and human resources, Legal, Training, Data, Evaluation and monitoring, Maintenance. Ethics emerged as a twelfth concept through qualitative analysis of the interviews. This study illustrates the inherent challenges and useful strategies in implementing AI in healthcare practice. Future research should explore various aspects of leadership, collaboration and contracts among key stakeholders, legal strategies surrounding clinicians’ liability, solutions to ethical dilemmas, infrastructure for efficient integration of AI in workflows, and define decision points in the implementation process.

## Introduction

The rapid progress of artificial intelligence (AI) has opened opportunities for its integration across diverse industries, including healthcare. AI technologies hold great promise for improving healthcare outcomes, enhancing patient care, and optimizing resource allocation [1]. Nonetheless, integrating AI into healthcare settings remains a challenge even with its substantial potential benefits [2,3]. It is, therefore, crucial to identify and address the barriers that hinder its successful implementation to fully harness the transformative power of AI in healthcare.

One of the primary challenges in AI implementation in healthcare is the complexity and diversity of healthcare systems [4,5]. Healthcare organizations often have fragmented data systems, making it difficult to collect, store, and analyze the vast amount of data required for AI algorithms [6]. Additionally, interoperability issues and data privacy concerns pose significant barriers to the integration of AI systems across different healthcare settings [7]. The heterogeneity of healthcare data also presents challenges in training AI models that can generalize across diverse patient populations, healthcare contexts and organizations [8].

Another key challenge is the need for intelligible and coherent implementation frameworks and guidelines [3]. While AI systems have shown promise in research and development settings, their translation into routine clinical practice requires robust frameworks that address technical, ethical, and regulatory considerations [4]. These frameworks should provide guidance on data governance, algorithm validation, patient privacy protection, and the integration of AI into existing healthcare workflows [3,4]. Without such frameworks, healthcare organizations may face uncertainty and hesitation in adopting AI systems, hindering their potential benefits.

Furthermore, the successful implementation of AI in healthcare necessitates a shift towards more information-driven ways of working. Traditionally, healthcare decision-making has relied heavily on evidence supporting current practice and the exclusive expertise and intuition of healthcare professionals [9]. The integration of AI systems challenges this paradigm by introducing data-driven insights and recommendations [2,10]. This transition requires changes in organizational culture, workforce skills, and workflows. Healthcare professionals need to be educated and trained to interpret and utilize AI outputs effectively, fostering a collaborative relationship between human experts and AI systems [2,11]. Moreover, ensuring the ethical and responsible use of AI in healthcare requires clear governance mechanisms and policies that address issues such as bias, transparency, and accountability [12].

The potential value created from successful AI implementation in healthcare is substantial. By leveraging AI technologies, healthcare systems can enhance patient outcomes, improve resource allocation, reduce medical errors, and increase operational efficiency [8,11]. AI-enabled decision support systems can aid healthcare professionals in making accurate diagnoses, predicting patient outcomes, and designing personalized treatment plans. Moreover, the integration of AI into healthcare systems can generate valuable insights from the vast amount of healthcare data, facilitating evidence-based decision-making and enabling proactive and preventive healthcare practices [1].

A thorough understanding of existing barriers to AI implementation within specific healthcare settings, coupled with effective strategies to overcome them, could significantly contribute to successful implementation. The concept of ‘implementation’ refers to an intentional effort aimed at adopting and integrating interventions into established routines [3]. A ‘barrier’ refers to an external or internal determinant that hinders such implementation efforts, while a ‘strategy’ facilitates and accelerates these efforts. This research seeks to provide a solid foundation for future studies and initiatives aimed at succeeding with implementation of AI in healthcare, realizing its full potential of transforming healthcare delivery and improving patient outcomes. Understanding the barriers and strategies is essential for several reasons. It allows researchers and practitioners to gain insights into the complex dynamics that influence the adoption and integration of AI systems in healthcare settings. It also supports determining common implementation challenges and designing necessary actions to address potential barriers, ranging from technical limitations to regulatory hurdles, ethical concerns, and workforce issues [2]. It could also contribute to methodological advancements in AI implementation, providing valuable insights and guidance for future implementation initiatives, informing researchers, policymakers, and healthcare professionals on the most effective ways to integrate AI systems into healthcare systems [4].

Based on the gap of knowledge described above, the aim of this study was to create a detailed inventory of barriers to and strategies for AI implementation in healthcare to support advancements in methods and processes, facilitating feasible implementation in healthcare settings.

## Methods

### Setting

The present study has been conducted as part of the research program “Toward Successful Implementation of Artificial Intelligence in Health Care Practice” [4] and with the overall purpose of developing a theoretically and empirically informed framework for AI implementation in healthcare that can be used to facilitate AI implementation in routine healthcare practice [13]. The first step towards this ambition is to review existing knowledge from scoping reviews and literature reviews on barriers to and strategies for AI implementation and thereafter to use qualitative interviews and an abductive approach to examine, complement and deepen the findings as well as to identify further knowledge gaps. Through these steps, the framework for guiding the implementation of AI systems in healthcare will draw on knowledge acquired from both literature and the expertise and experience of stakeholders and intended users of the framework in healthcare.

### Design

The study was inspired by a sequential explanatory mixed methods design [14]—had a sequential approach in employing different methods used to explain one another. The first part used principles of the overview of reviews research methodology [15,16], allowing integration of information from several systematic reviews to produce a comprehensive synthesis of evidence as the foundation for practical guidelines to be applied in practice. This design also allows inclusion and analysis of the primary studies contained within the included reviews [15]. The second part was a direct content analysis of empirical stakeholder interviews [17] building on the initial analysis of the literature and confirming relevance and supplementing results to the initial analysis of the literature.

### Data collection

#### Literature review

To identify scoping and systematic literature reviews, we searched the PubMed database using the following search string:

("scoping review" OR "systematic review") AND ("AI" OR "Artificial intelligence" OR "machine learning" OR "deep learning" OR "neural network") AND ("health" OR "healthcare") AND ("implementation").

The search was delimited to the most recent reviews published in 2020–2023. The search yielded 235 results. The inclusion criteria were: scoping or systematic literature reviews focused on empirical studies of AI applied in healthcare contexts and dealing with implementation in practice. Eight articles fulfilled the inclusion criteria after a review of the abstracts and were read in full text. Six reviews corresponded with the inclusion criteria and were included in this study, and of these, four were scoping reviews [18–21] and two systematic literature reviews [22,23].

Next, data was extracted from the included articles. However, this exercise appeared insufficient to fulfil the purpose of this study. First, these reviews [18,20–23] had an overarching focus on mapping the field of AI implementation or providing an overview of the specific machine learning models. Data on barriers and strategies provided in the review articles was brief and insufficient to correspond to the purpose of the present study. Second, the included primary studies had varying research designs and did not only include empirical studies of AI implementation in practice. It was therefore deemed impossible to derive which knowledge in the reviews specifically originated from empirical work based on AI implementation [20–23] as per the purpose of the present study. Third, the taxonomies of barriers and strategies differed among all the studies, as demonstrated in S1 Appendix. Based on these reasons, it was deemed impossible to extract all the information from the reviews. Instead, the focus of the analysis transitioned to the primary studies contained within the included reviews, as suggested by [15]. This resulted in the inclusion of a total of 193 papers from the reviews discussing AI implementation in healthcare. The sample consisted of 174 papers for review after removal of the duplicates (n = 19). To select the final sample for analysis, the following inclusion criteria were used: empirical study design, focused on AI implementation in routine healthcare practice, and published between 2011–2023. Exclusion criteria were: a study design of proof-of-concept, feasibility, or validation or if the study had not provided any insight into implementation (for example, focused only on clinical outcomes as a result of an implemented AI system). Full-text revision applying these criteria yielded the final sample of 38 papers for data analysis.

#### Interviews

In total 69 individual interviews were performed with professionals representing leader roles in general healthcare management (referred to LDR in the results, n = 26), organizational units participating in AI development projects and interviewed before implementation (cardiology unit referred to CRD in the result and emergency unit referred to EMU in the result, n = 30), and a primary care unit referred to PCU in the result which had recently implemented an AI system and uses it in practice (n = 13). These types of respondents were chosen to cover a life cycle of AI: from development projects to post-implementation phase, including the leadership’s perspectives. The interviews took place between the years 2020 and 2023 and addressed interviewees’ experience or perception of technology and AI systems’ implementations, considerations of possible effects of AI systems on patient, professional, and organizational levels, and success factors of and barriers to implementation. The interview guide is provided in S2 Appendix.

### Data analysis

#### Literature review

Data analysis aimed to extract reported barriers to and strategies for AI implementation from the individual papers (n = 38). The papers were read through and text describing a barrier, or a strategy was extracted and inserted into an Excel file. In the first step of data analysis, the data were deductively coded against three phases of implementation, namely planning, implementing, or sustaining the use [24]. The ‘planning’ phase referred to the organization’s preparatory activities for designing or selecting the AI system and evaluating the change it could potentially cause in the local context. The ‘implementing’ phase referred to the actual change project of integrating an AI system into practice. The ‘sustaining the use’ phase referred to the post-implementation efforts to ensure the sustained use of the implemented AI system in routine practice. The barriers and strategies were then inductively coded by one researcher (MN) using a thematic coding technique [25] and without a pre-defined index of the concepts. The concepts were discussed and reviewed by all co-authors. Disparities in opinions concerned whether a barrier could be reformulated in an opposite manner and become a strategy, i.e. a strategy to overcome a barrier (similar observation by [19]), or what kind of concept name would fit the best. The disparities were resolved in a consensus after discussions. A comparison of the concepts used in the present study and the analyzed scoping and literature reviews is provided in S1 Appendix.

#### Interviews

A direct qualitative content analysis was conducted [17] based on the concepts identified in the first step. The analysis intended to either confirm the previously identified concepts or to form new concepts.

## Results

The detailed inventory of barriers to and strategies for AI implementation resulted in 11 initial concepts based on the literature review: leadership, change management, buy-in, engagement, workflow, finance and human resources, legal, data, training, evaluation and monitoring, and maintenance. The following direct content analysis of empirical stakeholder interviews [17] added Ethics as an additional concept. Table 1 provides a list of concepts drawn from the analysis.

**Table 1:** Concepts* drawn out from the empirical studies and the interviews.

	Leadership	Change management	Buy-in	Engagement	Workflow	Finance and Human Resources	Legal	Data	Training	Evaluation and monitoring	Maintenance	Ethics
Sukums et al. [26]	B	S	B			B			B			
Joerin et al. [27]	S			S						S		
McCoy and Das [28]	S	S			S				S	S		
Petitgand et al. [29]	S				B							
Sun [30]	S	S		S		B			U			
Goncalves et al. [31]		S		S					S	S		
Sendak et al. [32]		S		S	U				S		S	
Lai et al. [33]		S										
Chong et al. [34]		S										
Moorman et al. [35]		S			U							
Strohm et al. [36]		S	B		B	U	B					
Lacey et al. [37]		S	B							S		
Litvin et al. [38]		S	B	S	S				S			
Sendak et al. [39]		S		S	S	S			S		S	
Ng and Tan [40]			B	S								
Reis et al. [41]			B	S			B					
Sandhu et al. [42]			B	S		S			B	S		
Baxter et al. [43]			B	B		B						
Rath et al. [44]			B									
Davis et al. [45]			S		S					S		
Wijnhoven et al. [46]			B	B		B	B					
Schuh et al. [47]			B			B	B	B				
Romero-Brufau et al. [48]			B		U							
Romero-Brufau et al. [49]			B		S	S		B				
Schlicher et al. [50]				S								
Murphree et al. [51]			S	S	S							
Anand et al. [52]				S								
Chonde et al. [53]				S						S		
Cruz et al. [54]				S						S		
Saverino et al. [55]				S		B						
Lee et al. [56]					S	S		B		S		
Moon et al. [57]					S							
Wen et al. [58]						U		B				
Morales et al. [59]						B						
Herman et al. [60]								B				
Xu et al. [61]				S	S			S				
**Interviews**	C	C	C	C	C	C	C	C	C	C	C	N

*B–a study discussed barriers related to the concept; S–a study discussed strategies related to the concept; U–a study discussed both barriers and strategies related to the concept; C–a concept was confirmed for relevance in the interviews; N–a concept was newly identified through the interviews.

**Fig 1:**
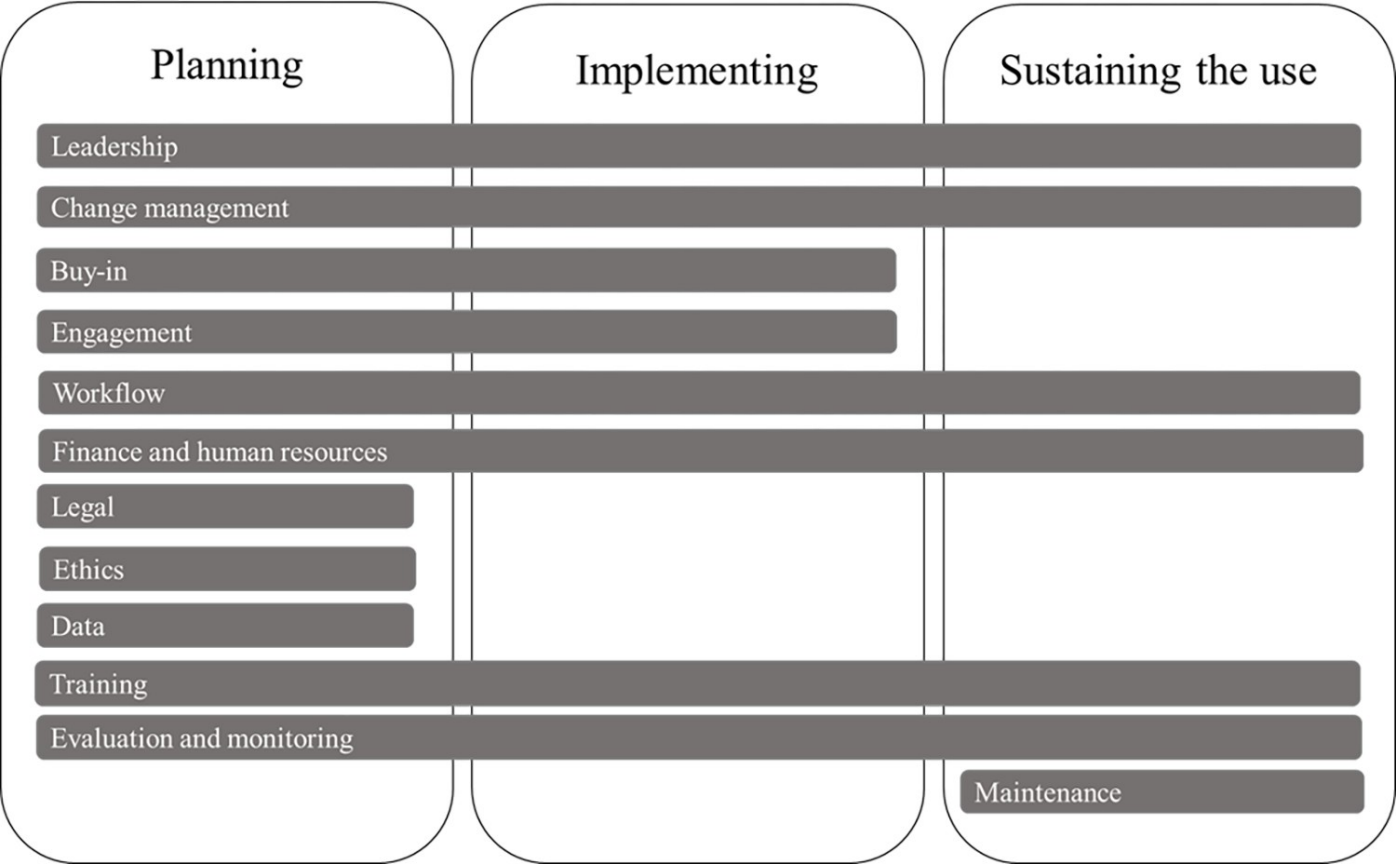
Concepts discerned from empirical studies and interviews related to the different implementation phases.

Based on the extracted barriers and strategies, the activities and approaches under some concepts are relevant in a particular phase of implementation, while others need to be addressed throughout the entire lifecycle of the implementation process; from planning to sustaining the use of the AI system [24], as shown in Fig 1. The result includes barriers to and strategies for AI implementation in healthcare in relation to each of the 12 concepts as well as to the implementation phases.

### Leadership

‘Leadership’ refers in this study to approaches or activities performed by healthcare leaders to promote and support the change due to AI’s implementation.

#### Barriers

Implementation suffers from a lack of leadership and guidance from supervisors [26].

#### Strategies

When planning an AI implementation, senior leadership should set a common vision among the stakeholders [27–29] (CRD). Leaders need to be involved as stakeholders and should be informed that they need to provide strong support to the implementation team throughout the implementation (PCU). When implementing the AI system and when aiming to sustain its use, hospital managers can employ their formal power to establish follow-up procedures (e.g. weekly meetings) on the utilization of AI [30]. The leader in charge of the implementation should have both clinical and IT background (CRD).

### Change management

‘Change management’ in this study refers to activities dedicated to promoting change in practice and supporting AI’s adoption and sustained use.

#### Barriers

Change management can stall if one assumes that change is wanted. Healthcare professionals have intense work schedules, become close friends over the years, they protect each other and might have reasons for not favoring the upcoming change (LDR).

#### Strategies

Communication is an essential part of change management during the planning of implementation (PCU). It raises initial awareness among staff about the upcoming change due to the AI system and its potential impact on processes [31] (CRD). In some cases, a cultural shift will be required to accommodate the new practice that would be formed due to the introduction of AI (e.g. working in a more predictive and proactive than reactive manner) (LDR). Communication should focus on a vision for change that needs to be communicated to all relevant stakeholders and organizational units [32]. Communication in forms of periodic meetings and newsletters should arouse curiosity, create a sense of urgency to improve care and highlight which potential benefits can be expected from the AI system [31] (LDR), which can be even further emphasized if external conditions such as a global pandemic reinforce the change [33].

Middle managers have a great influence on how the issue is driven and decisions are taken locally, therefore, ensuring their engagement and commitment is vital (LDR, PCU). They could be the closest go-to people for healthcare professionals who possess concerns about AI and the upcoming change (PCU). The middle management could become engaged if they understood what the AI system is about, how it works and in what way it should be used. After getting them on board, it is easier to initiate and ensure participation in the voluntary pilots (PCU). Local ‘champions’ (a dedicated group) who were involved in the design and development could be appointed to promote the use of AI among colleagues [32,34–36] (PCU). More healthcare professionals could organically be added to this group to diffuse the new practice (PCU). In addition, training new skills is closely intertwined with the success of change management, for healthcare professionals to have ability to evolve together with the change and to not fall into the old ways (PCU).

Informal communication between healthcare professionals and AI developers should be promoted during implementation to create trust in AI [30] and to help understand the value of outputs better [32]. Several weeks could be dedicated to strengthening relationships and building communication channels prior to the launch of the AI system [32].

To sustain the use of AI, clinicians could be incentivized (e.g. performance-based incentive schemes) for using an AI system [26]. In addition, gamification strategies could be implemented in the use of technology to create a sense of competition and potential rewards among the users [34,37].

Continuous monitoring of the actual use of AI systems is another important strategy that can enforce change [30,38], especially when the old routines are not allowed [38] (CRD). This can be achieved through the specially formed bodies of staff to monitor that the change should take place, e.g., quality improvement teams including clinicians, administrators, and technical staff [28], or the monitoring team responsible for the AI system to be operational most of the time [39].

### Buy-in

‘Buy-in’ in this study refers to various aspects that can affect the user’s perception regarding the new practice of using AI and can lead to acceptance, trust, and willingness to adopt and use the system in daily practice.

#### Barriers

Healthcare professionals may worry that new workflows or new set-ups that are formed due to the planned use of AI can potentially reduce their esteem and social status [40,41] or might even endanger their identity as medical professionals [41] (LDR, PCU, EMU). Further, healthcare professionals can be worried that an AI system would gradually make them dependent on technology and spend less time with the patient thus insufficiently utilizing the human interaction eventually delivering less care which would affect patients’ trust in healthcare provided by humans [41,42] (LDR, PCU, CRD).

Concerns have also been raised for the risk of creating an unequal care situation if the AI system is not uniformly adopted across the organization leading to larger differences in the care delivered by different professionals and potentially work duplication (LDR). Several studies have found that clinicians believe that human physicians surpass technology when it comes to intuition and subjective assessment, and that clinical judgement by human physicians is superior to algorithms [38,41,43,44] (LDR). For example, they are afraid to miss out on unusual events (due to data bias) when the healthcare professional’s role is to rule out dangerous events and, if necessary, to initiate a referral (EMU). However, they recognize that AI-based decision support systems can be useful for clinicians with less experience or in hospitals with resource constraints [42].

Further, a question of ‘what is the added value of AI?’ is relevant [36,37,45,46] (EMU). Sometimes, it can be troublesome to objectively evaluate the opportunity of using AI in a clinical context due to a lack of understanding of the AI model, its conclusions (e.g., a score), or a lack of clear use cases [26,42,43,47] (EMU, CRD). This can lead to the perception that the current care is optimal and there is no need to introduce AI [38,44,47].

Clinicians might experience fatigue if several models are being tested or multiple improvement initiatives are being conducted in an organization [43]. Clinicians also experience “alert fatigue”, which denotes inadequate responsiveness to the alerts due to increased workload or frequency of alerts. A need for an action to dismiss an alert can burden clinicians. Another example is when false positives or false negatives are frequent, and it can lead to stopping using the AI system [32,48] (LDR, EMU, CRD). Clinicians can also become model-averse when they suspect that developers of the solution lacked domain knowledge and had wrong assumptions [24,47] or integrated it poorly into the existing workflows and IT systems [37,41,48,49].

Creating conditions for healthcare professionals to try using an AI system, test its quality and see its results can facilitate their buy-in (PCU). However, experienced technical aspects of AI systems also affect the buy-in by staff such as mistakes in the algorithm when they try it out [41]. Slow AI systems would not be helpful in busy schedules [26], which could be applicable to the software, hardware, and network [38]. Finally, if AI systems are not in a physical space that is the most relevant in the workflow, it would not be convenient to use and require extra effort and resources ultimately affecting the buy-in and actual use [38].

Usability aspects are also important for the buy-in. Barriers are created when AI systems are less intuitive [24,38] (LDR, PCU, CRD) and when interface design and model output format are poorly tailored to future users [28].

#### Strategies

The buy-in and stakeholder support across the organization are necessary throughout the AI implementation project lifecycle, but the foundation of the buy-in should be in place when planning an implementation which benefits from acknowledging that AI causes concerns among healthcare professionals and general society (LDR, PCU). The buy-in from the leadership and managers creates conditions for the buy-in from healthcare professionals if the managers understand the system’s purpose, way of using it, and giving approval and support to proceed with introducing it to the employees (PCU). Furthermore, conditions for a strong buy-in are created when the request for developing an AI solution is initiated by local clinicians [47]. Feeling the local importance of the problem and the necessity to solve it creates better chances that healthcare professionals could achieve buy-in and promote the AI project and system to their peers [32] (LDR). If an AI system concerns an interaction with patients, investigating the interests of patients in using such a system is recommended (PCU).

Clinicians might be more comfortable with an AI system during the implementation if they are given power to decide on the model performance parameters [32], if the boundaries of clinicians’ liability when using AI and whether or not the final decision relies on the clinician are clear (PCU). Building the AI development projects on the hospital’s locally defined problems, local data, and considering experiences by its personnel is key for the staff’s buy-in [39]. Buy-in is also supported when clinicians feel safe that the AI system can be ‘snoozed’ or turned-off if the goals are not achieved [28,39] (CRD). For example, clinicians would appreciate the possibility to choose to see a notification when AI based output is available, compared to automatically popping up notifications (LDR).

Further, sometimes it can be worthwhile to first introduce a project with a technically simpler ambition which is backed by published evidence [24]. Demonstrating the impact of such a project would likely provide trust and willingness to take part in further advancements. Furthermore, studies have reported that the technological features of the AI system are not sufficient to create trust and willingness to use the technology in practice [32,39]. Trust can instead be built by demonstrating progress when solving a clinical problem rather than showing the progress of developing the technology [39], by transparently informing staff how the system is built, how it works, what is the development plan over time, who takes responsibility, and what are the interests of different parties (PCU). It can then be worth arranging a peer-review of the AI model and problem definition by other institutions for extra verification that the assumptions are right, and the model can outperform clinical scores [39]. Trust can also be cultivated by creating possibilities for staff to try out the model [39] (PCU).

Communication about the utility of the AI system is another way to enhance trust and buy-in, but the communication needs to be adjusted and address value that is meaningful to different types of stakeholders [39] (CRD). It is crucial to tailor the amount and type of evidence based on relevance when communicating about the model; patient outcomes are most interesting to physicians, while numbers and statistics to administration and managers [32]. Also, framing of messages about AI matter–some cases report not mentioning AI at all, but for example framing it as a support tool [32] or emphasizing it as a way to improve quality and complement care or even provide an extra ‘opinion’ where disagreements or ethical dilemmas occur (LDR). It is recommended to emphasize the evidence’s transparency and strength in cases where scientific evidence about the AI model is published [35]. Furthermore, stakeholders appreciate clearly presented information about the indications and contraindications of the model, demonstrating awareness of its strengths and weaknesses. An example of such a presentation could be a “Model fact sheet” as described by [39].

### Engagement

‘Engagement’ in this study refers to involving stakeholders in different activities throughout the phases of AI implementation to contextualize and ensure the innovation’s fit to the organization.

#### Barriers

Engagement suffers when communication, socialization, and alignment among different relevant stakeholders are insufficiently organized and promoted. This leads to a limited understanding of stakeholder needs, perspectives, and will complicate the problem assessment, which is especially important in the planning phase of implementation [32,43,46]. Engagement can be inhibited by the stakeholders’ skepticism from the start of AI implementation (for example, due to overload, a multitude of information systems or other), which can turn into a barrier to creating productive partnerships that could facilitate the implementation [24,38,50] (PCU).

#### Strategies

Engagement of stakeholders is necessary for building partnerships and relationships that can pave the way for trust and adoption of the technology. Stakeholders should be involved as early as possible, from the planning of the implementation process [31,39–41]. The group should be inter-disciplinary, and it is especially important involving clinicians, potential users of AI, hospital leaders, quality or process improvement units, human resources, and IT units [24,32,42,51–53] (LDR, PCU).

Building partnerships with key members of staff inside the hospital is key at the onset of the project [24,51] (PCU). It can allow for better problem definition, can facilitate the engagement of staff, and gain political support. The partners can be useful for their credibility and experience in solving clinical problems and will bring in their trusted networks [39]. To create conditions for better support, the project should align with the hospital leadership’s priorities [24] (PCU). Some studies have found it useful engaging stakeholders especially interested in analytics to promote the engagement of others in the implementation and to help translate the AI-delivered insights into practice [54]. Engaging the stakeholders allows aligning the project to the hospital’s priorities and preventing duplication of the initiatives and ensuring compliance with internal data and security policies.

Engagement of multi-disciplinary actors during implementation helps in co-creating AI solutions, services and workflows that align with the context and needs of the patients and caregivers, and in performing validation studies and receiving feedback [24,27,31,46,53]. Common activities could start with meetings and analysis to understand a problem and complexity, listing requirements, creating a shared vision for the implementation, and mapping the preliminary workflow [32,40]. Regular meetings between clinicians and developers are recommended with a goal of revising and contextualizing the model, defining the population parameters for training the model, ensuring a smooth integration of the tool in practice, and deciding how it would be evaluated [24,39]. Clinicians could also be involved in training the AI model and contributing to the user experience [30–31,41,55]. Involving clinicians is also useful to prepare them for training other staff members about the model—a capability that could be gained through accumulated intuition and experience from participating in the model development [39,55].

### Workflow

‘Workflow’ in this study refers to processes of standard care and new processes engineered due to the implementation of AI.

#### Barriers

Problems occur when the changes in workflows and potential conflicts in key performance indicators by different units are not carefully anticipated during the planning of implementation (CRD). The more changes in a workflow an AI system requires, the more challenges might be faced during its implementation. If the workflow changes significantly and clinicians need to adapt substantially, it could be perceived as additional work in the already loaded scope of duties and can hinder the implementation of AI [32,35,48] (PCU). For example, non-interoperable systems can lead to manual handling of data (e.g., printing the result of AI and carrying it further through the existing workflow), which burdens the activities [29] (LDR, PCU). Issues occur when there is no guidance for the stakeholders during and after the implementation, and they utilize the AI system in non-uniform ways. It becomes difficult to evaluate a system’s performance, and the intended benefits might not be attained and experienced and the utility of the system can become doubtful [36].

#### Strategies

The design of the new workflows during the planning of an implementation starts in the process of problem assessment [32,56]. It should be identified through collaboration in an interdisciplinary team, where in the process the problem occurs, who among the roles could benefit most from an improvement, and what changes are needed to attain those benefits. These insights can be achieved through data analysis and by observing or interviewing healthcare professionals [32] (PCU).

To address the feasibility of interoperability, an internal screening of relevant information systems deployed in the hospital and workflows related to the identified problem, is recommended: how are they currently solving this problem, what integrations with other systems and supporting infrastructure will be needed? It is important to evaluate whether the existing already contracted suppliers can join in solving the problem or an external collaboration should be initiated [32]. Interoperability between an AI system and electronic health record system (EHR) and other relevant systems at the hospital should be considered by an implementation team partnering with the technology suppliers [39]. Inter-operable AI systems have been shown to create conditions for efficiency of treatment and better clinical outcomes [51,57] (CRD).

Healthcare professionals should be included in determining the moment when an AI system is used and could bring the most benefit in the care process [32]. A prototype of the desired workflow, changes in the surrounding services and changes in related supporting infrastructure need to be designed, not forgetting decisions on the hardware, if necessary (e.g. a mobile phone or a tablet). A general insight is that the workflow, existing practice, current roles, and functions should remain as little disrupted as possible to fit the AI system [45]. Non-disruptiveness can be perceived as safer for patients [28], and it creates more chances for success in implementation [32,35,48]. It is important to consider that within the current practice, clinicians guarantee their professional accountability and trust with patients, and therefore, they can be protective of these workflows [39]. In addition, new roles and responsibilities need to be defined in relation to the new workflow [32]. It has also been demonstrated that the less the workflow involving AI goes across different units, the easier it is to manage the usage and chances of successful adoption increase [32].

The prototype of the workflow and the related infrastructure with AI should be iterated together with the healthcare professionals [24,32] (PCU). The prototype needs to be checked and tried out to identify how long the new process is, and what resources are, in fact, required to reach the benefits. For example, reviewing alerts might be time-consuming, which can delimit how many alerts can be reacted to over the day and affect the sustained use of the AI system [24,32]. The interface of AI systems needs to be customized to the workflow so that it is easy to use and sufficiently intuitive [49]. It is important to arrange regular meetings with senior leadership and related stakeholders to align on the unified view of the workflow after gathering the data on the performance metrics during the testing of the workflow [32].

To sustain the use of AI, performance metrics for monitoring the workflows and a plan for continuous improvements of the workflow need to be set [28].

#### Finance and human resources

‘Finance and human resources’ in this study refers to the funding and personnel needed to implement the AI system–from the early investment of these resources after the inception of the need to the funding needed for sustained use of the AI system and recruitment of staff.

#### Barriers

Clinical expertise is expensive, and engaging clinicians in planning and implementing AI projects results in cost in terms of time and money, which not all healthcare organizations can afford to spend [43,47,58] (LDR, PCU, CRD). In addition, a lack of published evidence of the value of some types of AI systems hinders attracting funding for initiating the implementation [36].

An increased use of human resources occurs during the implementation when the clinicians are insufficiently equipped with computers in the physical space where the AI system would bring benefits based on the workflow [55] (PCU). Underinvestment in physical infrastructure in hospitals can become a barrier to the sustained use of AI as it creates problems for inter-operability with other systems or increases demands for human resources (e.g., due to dual manual data entries together with digital ones) [26,30,46].

Other challenges relate to obtaining financial resources for commercializing, scaling, and sustaining the use of AI in organizations [59]. Substantial costs for regulatory approval of AI solutions relate to the creation of the documentation for demonstrating conformity, as well as for testing and quality control of the product. Not having this financing inhibits obtaining regulatory approval and slows down the exploitation of the solution commercially and scaling to other organizations. As a result, many AI solutions remain in the experimental state or in research projects that perform testing of the product in healthcare organizations with an ethical board approval but are not permitted to scale [47] (LDR).

#### Strategies

Budgeting recruitment and the involvement of several roles to actively engage in AI implementation during the planning of AI implementation has been reported as a strategy that contributes to successful implementation (LDR, CRD). Leaders are recommended to plan recruiting and involving trusted physicians [59], innovation managers [36], specially qualified full-time roles for the project to work cross-functionally with clinicians and other stakeholders during the implementation [39,42]. It is recommended to also budget for the preparation of the training materials around the AI system. Studies have indicated that this activity demanded substantial resources [39].

Financial sustainability of the AI use and continuous maintenance and improvement of the model could be resolved through fundraising [56] or public-private partnerships that have been proven a sustainable model to ensure financing [49].

#### Legal

‘Legal’ in this study refers to the legal framework surrounding AI systems in healthcare, local policies, and compliance with international and local law.

#### Barriers

Obtaining regulatory approval for ensuring dissemination and market utilization of an AI solution has been mentioned as a substantial resource burden for the creators of the software, delaying implementation. Further, procurement procedures create barriers for innovation to enter healthcare organizations (PCU). Many solutions do thus not reach actual adoption and sustained use in clinical practice [47]. For the AI solutions that are considered for implementation, an uncertain legal framework is a barrier when it comes to clinicians’ responsibility and liability in case of adverse events after relying on an AI algorithm in making decisions or ignoring it. It reduces clinicians’ motivation to implement AI and use it in the future [36,41,46] (LDR, EMU, CRD).

#### Strategies

Neither the empirical studies nor the interviews discussed strategies for overcoming legal issues related to the implementation of AI.

#### Ethics

‘Ethics’ in this study refers to the moral principles, values, and guidelines that govern the development, deployment, and use of AI technologies to ensure that these systems are designed and utilized in a responsible, fair, transparent, and accountable manner.

#### Barriers

Healthcare professionals are concerned that AI would impersonalize care by relying on other patients’ data used in training the algorithm (LDR). Another concern is that sometimes the patients do not want to know the predicted outcomes, such as an increased risk of a serious condition or even predicted death (LDR, EMU).

#### Strategies

AI can be seen and promoted as a helpful assistant in approaching ethical dilemmas in care, such as discharging a patient, suggesting palliative care, and similar, building on an argument that AI can provide more individualized recommendations based on similar patients than clinical guidelines that are based on a group level (LDR). Further, it is considered ethical to be transparent to the patients that they interact with AI, not real humans (PCU). Patients might be extra cautious in accepting recommendations by AI when these concern more serious interventions (e.g. surgical) and might wish to have a second assessment by a human clinician (LDR). Lastly, a possibility for the patients to choose how much personal data to share with AI to be able to make an assessment is desired (LDR).

#### Data

‘Data’ in this study refers to all the aspects directly connected to the data that is used to train the AI model; data quality and limitations, its availability and access, and data management and interoperability across different information systems.

#### Barriers

Insufficient data quality, such as unstructured and incomplete data, varying formats, labels and identifiers, insufficient frequency of data updates for real-time predictions, old data, low amount of data on specific patient types, and wrong medication lists create challenges in building high-quality AI models and maintaining them [47,49,56,57] (LDR, CRD). In addition, data might face reliability issues in the way it was created, for example, due to variations in handling individual cases in the workflows that generate these data. Even the key outcome indicator can sometimes not be estimated or predicted due to non-available or low-quality data [56]. Bias in the data sets is another concern under-representing patients from racial, gender, ethnicity, age and other related perspectives (LDR). Another barrier is the complicated access to necessary amounts of data due to legal and organizational boundaries [58,60]. Hospitals’ EHR does sometimes not support data processing and analytics so that the AI model can be implemented [24].

#### Strategies

Good quality of data is a prerequisite for a good AI system, and it has relevance through all the phases of AI implementation, but it is especially important in the planning phase when the key decisions regarding the model are taking place [56]. Data quality can be improved by training staff regarding proper data entries and maintenance and introducing data cleaning procedures [61]. The availability of rich data sets at the hospital for training the model is also a facilitating factor for AI implementation since it helps to train and keep updating the AI model [56]. When defining data sources to build the AI model on, it is important to have knowledge about how long the data was stored, how changes were made, and similar (LDR). An external company for analytics can be included when the hospitals’ EHR does not support the data processing and analytics [24].

#### Training

‘Training’ in this study represents education, professional skills and activities related to development of these aspects.

#### Barriers

The disparate extent of knowledge and skills among potential users and stakeholders can affect the levels of use of AI systems, and this needs to be taken into consideration when planning the implementation [30]. A lack of IT skills can especially affect the use of AI [26] (LDR, PCU). This may lead to misunderstanding of the AI model that conditions confidence in using its output for clinical decisions [42].

#### Strategies

Training for the target group needs to be organized during implementation in order to ensure the capabilities of using an AI system and to understand it (LDR, CRD). It can be arranged as a formal activity but can also take place informally and can happen during and after implementation [26,30]. Training can be conducted by a quality improvement team, an innovation team, AI developer, or through combined efforts between the hospital and the AI developer [28,30] (PCU). The focus of the training should be on how to understand the outputs of the AI system and how to act in the new workflows (LDR). It should be sufficient to tackle the ‘black box’ issue, although detailed explanations of the algorithm’s mechanisms may be avoided (LDR). Different stakeholders need to be included in creating the training materials. This will help make decisions regarding the workflow, and better understand the needs for staff recruitment in using the AI system [28]. The training materials should then be reviewed by several stakeholders (e.g., nurses) who were involved in the development of AI systems and have the necessary expertise [39].

Clinicians having a trainer role should be trained to understand the hardware and software of the AI system and how the algorithm functions, the change management processes, and how to interact with the clinical teams in the trainer’s role [31] (LDR). The staff who perform the training also need to develop good listening skills, empathy, and technical understanding about the AI system [31].

An additional strategy to support training activities and to increase healthcare professionals’ understanding of AI and its uptake is to create social media channels where IT staff could share AI-related knowledge to healthcare professionals [32].

The post-implementation training could take a format of individual training (e.g., during on-boarding of a new employee and one-on-one training sessions during shifts of the staff have been proven a good practice [38,39], but also peer-group meetings and workshops using role plays (a patient and a doctor) have been found useful where users could discuss best practices [38] (PCU). An AI test environment for continuous onboarding of healthcare professionals is recommended in order to have a safe space to experiment with AI and to ask questions (LDR).

#### Evaluation and monitoring

‘Evaluation and monitoring’ in this study refer to activities related to understanding the worth of AI for practice, developing knowledge, researching whether practice has improved and monitoring compliance. These activities should guide decision-makers in matters of investing resources, adopting, and sustaining the AI system in routine care.

#### Barriers

Neither the empirical studies nor the interviews discussed barriers to evaluation and monitoring related to the implementation of AI.

#### Strategies

Implementation and investments in deploying AI systems are more likely to occur and be sustained if the project has demonstrated an impact [24,45,57]. Planning an evaluation is, therefore, imperative (CRD). Several strategies for clinical assessment are proposed. For example, a pilot intervention based on the AI system and new workflow could be implemented for just one service, one population, limited AI system’s functions, or one organizational unit at a time, and the standard care would serve as a control [24] (LDR, PCU, CRD). Taking baseline measurements and planning adequate control and randomization components are recommended when useful [28]. Assessing patient safety through a risk and impact analysis should not be forgotten when planning an evaluation (LDR). Cost and benefit evaluation is also important information for decision making whether to adopt an AI system (LDR). After the testing period, clinicians would have familiarity with the AI system and might be able to determine whether the system could be useful in their routine care which can serve in further assessments (PCU).

Another strategy is to test and monitor clinicians’ adherence to an AI system and new clinical workflows. Assessing adherence can lead to useful insights into additional measures that could improve adherence rates. Adherence evaluation should be conducted once more after implementing these measures in order to verify the effectiveness of the measures [54].

Simple quantitative measures can also be observed and have a convincing effect. For example, how many more potentially dangerous cases were identified by the AI system that were missed by professionals [42]. Qualitative results (e.g., user experience, feedback) from a trial period, together with quantitative measures, should be collected to evaluate the most impactful and useful components of the AI system and to initiate the modifications [27,61]. Such feedback can be collected by giving temporary access to a limited group of clinical professionals who would try using the AI system [27].

To sustain the use, quality improvement methods such as the Plan-Do-Study-Act cycle could be useful for obtaining regular, continuous feedback collection (could be automated) about the AI system in the clinical context [28,37,53,56] (LDR). For example, a special team can proactively visit users, listen to them with empathy and respect, provide hands-on support with the AI system or the workflow, collect feedback for improvement and training needs [31]. Organizing assessment sessions involving the AI developers is recommended to assess how the AI system works and problems with it, and to reflect about the workflow and organizational issues like staffing affects the use and usefulness of the platform (PCU).

#### Maintenance

‘Maintenance’ in this study refers to new organizational units, bodies, or practices formed due to AI implementation in practice and aimed at supporting its sustained use.

#### Barriers

Neither the empirical studies nor the interviews discussed barriers to establishing new organizational structures related to the implementation of AI.

### Strategies

Sustaining the use of AI in the organization can be reinforced by creating a support system after the implementation. Suitable strategies to reinforce this support system is organizing information meetings together with AI developers (PCU, CRD), super-user meetings to have an in-depth discussion on the system’s functionalities and possibilities (PCU), weekly and later monthly follow-ups to check what works well and not, and to identify what needs to be changed (PCU). An internal service desk handling deviation reports and answering questions (LDR, CRD, PCU) and a dedicated contact person from the AI developer side who could be contacted any time for reporting faults and asking questions is a valuable bridge supporting clinicians (LDR, PCU). It is important to monitor how the faults have been resolved, since it can be demotivating to see repeating mistakes in the system (CRD). Another strategy is to establish a cross-functional governance committee for AI implementation, which is recommended to meet monthly. It could include professionals from the closest to the patient (nurses, physicians) to innovation managers and leaders of the organization. The committee’s goals could include AI’s usage promotion, training new users in terms of application and workflow, tracking effectiveness and compliance, reporting, and planning financial sustainability for continuing using the AI system in the organization [32,39]. The agenda of such a committee could also include reviewing individual patient cases where the treatment had failed to increase learning [39]. Another establishment could be an external data safety monitoring board to monitor the safety and efficacy of an AI system. It could consist of relevant researchers and a clinical representative [39].

## Discussion

The primary objective of this study was to disseminate knowledge that could generate actionable insights and contribute to the methodological advancement within the realm of implementing AI systems in healthcare. By delving into the specifics of the implementation process, identifying barriers and strategies, and meticulously documenting these findings, a more nuanced perspective on the organization of AI implementations and associated considerations was achieved. This work represented a significant progression beyond previous systematic and scoping reviews focused on AI implementation [18–23] by shedding light on critical aspects essential for practical learning and methodological refinement in the domain of AI implementation. It is noteworthy that previous research has demonstrated that individual case studies tend to provide a limited viewpoint on AI implementation, often focusing on a specific scientific domain, utilizing varying terminologies, and emphasizing either barriers or strategies. This fragmentation complicates both inter-study comparisons and the accumulation of knowledge [19].

Additionally, in this study, we categorized the identified barriers and strategies related to AI implementation according to the various phases of the implementation process, ranging from initial planning to the ongoing maintenance of the AI system [24]. To the best of the authors’ knowledge, this represents the inaugural attempt to offer a process-oriented perspective as a supplementary asset to the inventory of barriers and strategies. Such an approach enables practitioners and researchers to engage in a more nuanced and comprehensive examination when tackling AI implementation challenges.

### Phases of AI implementation

#### Planning

A large variety of barriers and strategies were identified in relation to the planning phase, some of them particularly manifesting in AI implementations, and their summary under each of the concepts is provided in Table 2. Successful implementation of AI in healthcare requires extensive and thorough planning, as shown by the strategies reported in the empirical cases included in this study. Implementation of AI is enforced if the introduction of the AI system is part of a wider formal strategy (local or governmental) [36]. An absence of such strategies creates a dependency on organizational priorities and can inhibit innovation [59]. A sound needs assessment should be performed at the start of the planning to determine needs and where they occur [32,39]. Lessons from past implementations should be considered [39]. Engaging interdisciplinary stakeholders (patients and their representatives, clinicians, AI developers, and the government) is particularly useful in such an assessment and later phases of the implementation [24,32,42,51–53] (LDR, PCU). Considerations regarding ethical aspects of AI system and its implementation have not been identified when reviewing previous empirical literature but became apparent during the interviews with healthcare professionals and leaders. This finding demonstrates that combining study methods is a valuable strategy for obtaining a better understanding of barriers and strategies that healthcare actors are concerned about when implementing AI. These insights should feed into the design of an AI system or should inform a selection of commercially available AI systems regarding their fit with the local needs and workflows [24,27,31,46,53]. In addition to that, locally developed or commercial AI algorithms should undergo a careful assessment regarding data quality that AI model has used, data representativeness to the local population, assessment of algorithmic bias and accuracy [47,49,56,57] (LDR, CRD).

**Table 2:** Barriers and strategies in the planning phase of AI implementation.

Barriers for planning
**Leadership** • Lack of guidance and common vision among stakeholders **Change management** • Personal reasons by staff leading to resistance to change **Buy-in** • Concerns for self-esteem, social status, identity, gradual knowledge loss; Perceived irreplaceable experience, intuition, human judgement, potential unequal care; Not understanding value of AI, current care perceived as optimal. **Engagement** • Lack of communication, socialization and alignment among stakeholders; Stakeholders’ skepticism **Workflow** • Not anticipated conflicts between potential workflow changes and KPIs of units; Significant changes in workflow (disruption); Non-interoperable systems. **Finance and human resources** • Not affordable to dedicate clinicians’ time to implementation; Lack of published evidence on AI system hinders chances of funding; High costs for regulatory approval. **Legal** • Regulatory approval requirements; Procurement procedures; Uncertain legal framework for clinicians’ liability when using AI. **Ethics** • Concerns that AI would impersonalize care; Patients not willing to know predicted outcomes. **Data** • Insufficient quality of data; Variations in handling workflows that create data; Bias in data; Complicated access to data; EHR not supporting data processing and analytics. **Training** • Disparate extent of knowledge and skills (also IT skills) among potential users can affect the levels of use of AI and create misunderstanding of potential.
**Strategies for planning**
**Leadership** • Involved leaders providing a vision and strong support to an implementation team. **Buy-in** • Achieving buy-in by top management can positively affect buy-in by employees; A request for developing an AI solution is initiated by local clinicians (a local problem). **Change management** • Communicating a sense of urgency, vision for change across organization; Raising initial awareness among staff about upcoming change and potential impact on processes; Engaging middle managers and identifying local ‘champions’. **Engagement** • Early involvement of stakeholders (also the ones interested in analytics); Inter-disciplinary group; Alignment with organizational priorities; Building partnerships with key staff inside a hospital. **Workflow** • Problem assessment with interdisciplinary team; Assess feasibility for workflow and infrastructure changes, and interoperability with existing systems at the hospital; Assess if current suppliers could be used or new collaborations need to be initiated. **Finance and human resources** • Budgeting for recruitment, involvement of staff, and for preparation of training materials. **Ethics** • Promoting AI as an assistant in ethical dilemmas; Transparency with patients that they are interacting with AI; Integrating a possibility for patients to choose how much data they want to share with AI. **Data** • Training staff how to ensure data quality during entries; Introducing data cleaning procedures; When training the AI model, knowledge about how long the data was stored, how changes were made. **Training** • Planning for training, budgeting and planning of the preparation of training materials and of training activities. **Evaluation and monitoring** • Planning a pilot intervention on AI system and new workflow; Having randomization and control groups; Assessing patient safety; Cost and benefit evaluation; Adherence metrics.

Further, aiming for the stakeholders’ buy-in throughout the implementation is a continuous effort that helps achieve adoption, but AI brings a range of special challenges around trust. It is driven by various factors such as insufficient evidence of the AI systems’ value to the problem or a lack of use case, perceived or potential threats to clinicians or patients and ethical concerns, or insufficient IT literacy hindering an understanding of the AI model and its parameters [26,36,37,41–43,45–47] (EMU, LDR, PCU, CRD). Communication and training of clinical staff should be planned for tackling these challenges [32,39,43,46] (CRD). Clinicians’ liability is another key concern which, if not legally established, becomes a strong hinder to the buy-in (PCU). Leadership needs to set a vision, align the AI implementation initiative with the organizational priorities, and initiate communication focused on the identified need for change and possible added value for practice [27–29] (CRD). Middle level managers and leaders have to plan for the data, finance and human resources, which have been shown to strongly accelerate the implementation, while the lack of such resources reduces the chances of successful implementation [39,36,42,58]. In addition, it is recommended to consider how the AI system’s utility can be assessed [24,45,57], which changes in the hospital’s infrastructure would be required for the system’s inter-operability [32] (CRD), and how such a system can be financially sustained [49,56].

#### Implementing

A large variety of barriers and strategies were identified in relation to the implementing phase, and their summary under each of the concepts is provided in Table 3. Change management becomes a key factor during the implementation with communication between clinicians and developers being at the center in conditioning people’s engagement and acceptance of the change [30,32] (LDR, PCU). Framing of messages around AI is tricky and should take into account existing preconceptions and concerns around AI, knowledge gaps, as well as potential cognitive bias among the receivers [26,36,37,41–43,45–47] (EMU, LDR, PCU, CRD). Clinicians, together with developers, should take an active part in the system’s development and improvement activities, and in ensuring good usability of the system [24,27,31,53,61]. The implementation team needs to work towards developing the right AI system for the right problem, designing the new workflows and infrastructure for the system’s use and defining roles and ways of organizing to match the processes improved by the AI system [32,40] (PCU). Collaboration with external IT providers should be considered to fill the gaps in the infrastructure [32]. The new workflow should be iterated with the relevant staff to gather knowledge on the actual length of the process and resources required [32] (PCU). The buy-in by staff is dependent on evidence of an AI system’s impact on the problem-in-question [32,39]. An evaluation methodology should therefore be formulated, and the initial evidence (including adherence to using the AI system) provided using quantitative and qualitative methods [27,28,54]. Based on this information, leaders should communicate the added value of the system and adjust the message depending on the stakeholder type. Training materials should be defined, trainers’ roles assigned, and competences acquired during the implementation [28,31,32].

**Table 3:** Barriers and strategies in the implementing phase of AI implementation.

Barriers for implementing
**Buy-in** • Clinicians’ fatigue, alert fatigue, false positives, wrong assumptions and lack of domain knowledge seen in the model; Lack of technical quality and incorrect physical space of using the system, poor usability; Not seeing value in AI. **Workflow** • Lack of guidance on how to act in a new workflow–non-uniform usage; Non-interoperable systems. **Finance and human resources** • Underinvestment and lack of physical infrastructure to bring benefits of AI.
**Strategies for implementing**
**Leadership** • Establishing follow-up procedures for adherence and utilization during pilot. **Buy-in** • Clinicians decide on model performance parameters; Using local data and local experiences; Starting with a technically simpler project; Showing progress in solving a clinical problem; Arranging a peer-review of the AI model; Possibility for clinicians to try the model; Conveying value through communication using good-quality evidence, thoughtfully framing messages about AI system, exposing strengths and weaknesses, indications and contraindications, and clinicians’ responsibilities; Assurance for snoozing or turning-off the AI system if not satisfactory. **Change management** • Informal communication helping to convey value of AI outputs; Building communication channels and strengthening relationships. **Engagement** • Common meetings to understand problem, product and workflow requirements, to create a shared vision; Regular meetings between healthcare professionals and AI developers; Involving healthcare professionals in training AI model and user experience formation. **Workflow** • Determining a moment when AI system could be most useful; Designing a prototype of the new workflow and surrounding technical infrastructure–prioritizing less disruption; Estimating resources; Defining new roles and responsibilities; Iterating prototypes of workflow and infrastructure with relevant staff; Testing new workflow and collecting data on performance and usability; Customizing AI system’s interface to match the workflow; Arranging regular meetings with leadership and stakeholders to align on unified vision of the workflow. **Finance and human resources** • Performing fundraising or arranging public-private partnerships for ensuring possibilities for actual use of the system. **Training** • Doing training formally and informally; Focusing the training on how to understand the outputs of the AI system and how to act in the new workflows; Addressing ‘black box’ issue; Including different stakeholders in preparing training materials and reviewing them; Training clinicians to take on a trainer’s role from technical to empathy knowledge and skills; Creating social media channels for sharing knowledge. **Evaluation and monitoring** • Having a trial period; Taking baseline measurements; Collecting quantitative and qualitative data on user experience; Assessing clinicians’ adherence; Identifying most useful components of AI system.

#### Sustaining the use

A large variety of barriers and strategies were identified in relation to sustaining the use phase, and their summary under each of the concepts is provided in Table 4. The AI systems can deliver benefits to patients, clinicians, and the overall healthcare system only when their use is sustained. The current study has introduced a novel concept referred to as "maintenance,” as discussed in [32,39]). Interestingly, this concept had been largely overlooked in previous literature reviews. Two reviews [20,22] did include studies related to "maintenance” within their samples, but they did not explore this aspect (see S1 Appendix). Another review [18] briefly touched upon it within the broader concept of “Organizational efforts.” However, this characterization appears to be somewhat imprecise when compared to the essence of its meaning, which more accurately relates to the creation of new organizational forms and practices, such as a helpdesk, special committees, or units, specifically designed to support and sustain AI implementation efforts. However, the present study aligns with previous research [18] in acknowledging the importance of isolating this concept as distinct from discussions about workflow or resource allocation. This recognition underscores the significance of "maintenance" in the context of successful AI implementation. To maintain the use of the system, performance metrics for a workflow should be set and monitored [28]. Continuous improvement principles with control mechanisms and measurements should be applied incorporating the collection of personal reflections from involved stakeholders on the AI systems use in practice and on user feedback [24,30,37,53]. Based on the collected information, evidence of the AI system’s added value should be used in communication about the benefits, strengths, and weaknesses of the implemented system. To support the system’s utilization incentives for clinicians to sustain its use should be provided [24], continuous training and education of existing and new staff should be organized, making sure the turnover of staff does not hinder the use of the system [26,35]. Furthermore, establishing a support system for the sustained use of AI is essential (CRD, PCU). A helpdesk can address operational questions, and new organizational units such as a cross-functional governance committee can take on responsibility for tracking effectiveness and compliances, planning related resources, promoting AI’s usage, and ensuring learning from the experience of using the AI system [32,39] (PCU).

**Table 4:** Barriers and strategies in the sustaining the use phase of AI implementation.

Barriers for sustaining the use
• **Leadership** • Lack of sustained guidance, common vision among stakeholders **Workflow** • Lack of guidance on how to act in a new workflow–non-uniform usage **Finance and human resources** • Underinvestment and lack of physical infrastructure to bring benefits of AI. **Workflow** • Lack of guidance on how to act in a new workflow–non-uniform usage. **Finance and human resources** • Underinvestment and lack of physical infrastructure to bring benefits of AI.
**Strategies for sustaining the use**
**Leadership** • Establishing follow-up procedures for adherence and utilization during the actual use. **Change management** • Incentivizing clinicians for using AI (performance-based); Gamification and sense of competition; Continuous monitoring of AI usage enforces change, using old ways unacceptable. **Workflow** • Setting performance metrics for monitoring the workflow; Setting a plan for continuous improvements. **Finance and human resources** • Fundraising or public-private partnerships to secure funds for sustained use of AI. **Training** • Post-implementation training (individual and peer-group), role plays; AI test environment to experiment with the system and to ask questions. **Evaluation and monitoring** • Quality improvement methods; Plan-Do-Study-Act methodology; Collecting feedback continuously; Proactively visiting users; Organizing assessment sessions with AI developers. **Maintenance** • Information meetings together with AI developers; Super-user meetings; Weekly/monthly follow-ups to check how system works and needs for change; Internal service desk for handling faults and reports; Dedicated contact person from AI developer’s side; Cross-functional governance committee for AI usage promotion, training new users, tracking effectiveness and compliance; reporting, planning financing; External data safety monitoring board to monitor safety and efficacy of AI.

Finally, many of the identified barriers and strategies resembled the challenges and effective principles towards successful projects established in related domains such as IT project management, quality improvement, and change management. This validates that AI implementations largely share common traits with IT and quality improvement projects and that those methodologies should be incorporated in managing the AI implementations. For instance, ensuring support by leadership, creating a sense of urgency, or communicating throughout the project are well-known strategies in the change management field [62,63]. However, these strategies may have a nuanced application when it comes to AI, and it would be valuable to investigate them in-depth in future studies.

#### Identified gaps

Several gaps were identified where the empirical cases were not reported on in relation to the process of AI implementation and lessons learned. First, the studies failed to report on the actual activities of leaders when providing support to AI implementation initiatives. Earlier research has emphasized that leaders might lack expertise and awareness of AI-related challenges to be dealt with [2]. Such knowledge would allow leaders to plan and perform actions related to their own role, which is an enabling one [64,65]. Second, the empirical studies were deficient in explaining effective strategies for communication with staff to promote the intention and vision motivating the AI implementation. Previous research has shown that healthcare leaders find communication around AI complicated due to its conceptual ambiguity [66], it is therefore especially important to extract learning from practice; successful and unsuccessful. Third, the contractual relationships and collaborations between the hospital and AI developers or partner organizations (e.g. universities) and suppliers and how this collaboration can be navigated in terms of initiation, negotiation, procurement, deployment and post-deployment maintenance have not been elaborated on in the research knowledge. Such knowledge would help healthcare providers anticipate the complexity and feasibility of entering such collaborations. Fourth, significant legal questions in relation to liability by clinicians when using decision support systems are insufficiently covered–how hospitals create safety and confidence in using the decision support systems while the legal framework is yet to be defined. Earlier research has identified that unclear boundaries of professional liability and a lack of tools for a healthcare professional to self-protect can inhibit adoption of AI [67,68]. Fifth, ethical considerations have not been reported–which types of ethical dilemmas were considered, practically faced and how they were resolved throughout the implementation phases, a challenge informed by the previous research [69–72]. Sixth, the hospitals’ technical infrastructure and access to data–how hospitals need to be prepared for the AI system to be integrated smoothly and the related complexities. Previous research has emphasized that strategic development of internal IT infrastructure is a necessity for enabling AI systems’ integration into routine use [66]. Finally, research is lacking on the aspects of removing or “switching-off” the AI systems and the related impact of such a removal on healthcare organizations. On one hand, some studies mentioned that knowing that the AI system can be removed if it does not meet expectations provided comfort to clinicians and facilitated their buy-in [28,39]. On the other hand, there is a lack of knowledge on what the key decision points are (go or no-go) during the implementation process and how organizations and clinicians adapt to such a removal.

### Limitations

One limitation of the present study is that it may not have been able to include empirical articles on AI implementation in healthcare practice published in 2022–2023, if these were not incorporated in the scoping reviews or systematic literature reviews published in 2020–2023. This strategy has also delimited chances of capturing barriers and strategies attributable to the latest developments in the field of AI, such as generative AI and ChatGPT, and similar. Such interactive technologies might have added an additional set of complexity around data, buy-in and engagement when it comes to incorporation of this technology in healthcare settings. A separate search and inclusion of the most recent empirical studies could potentially add additional aspects to barriers or strategies. Furthermore, the study excluded articles that did not feature empirical case study research. Studies based on surveys or interviews could provide a complementary set of barriers and strategies and could be explored in future research. In addition, the study did not categorize and discuss barriers and strategies based on the type of AI model, the geography of implementation, clinical indication, or organizational unit. Also, the study focused on end-users as healthcare professionals and administrators without an explicit focus on patients as users of AI addressing their medical needs. An understanding of the barriers and strategies, particularly from the patients’ perspective, would possibly have provided additional dimensions to the results. All barriers and strategies were generalized, and hence, the contextual details of a barrier or a strategy are not provided. The variability in detail across the studies made it challenging to determine the completeness of the reported barriers and strategies in AI implementations, preventing a definitive priority ranking of strategies. Thus, we recommend healthcare organizations conduct their own assessments to tailor priorities to their unique contexts and goals. Finally, the present study did not aim at identifying the definite taxonomies of barriers and strategies to AI implementation, but given the advent of new data, the concepts used in this study might be revised in the future.

## Conclusions

This study aimed to create a detailed inventory of barriers and strategies in the different phases of AI implementation in healthcare in order to support methodological advancements on how to implement AI in healthcare settings. Building on empirical cases and interviews, barriers and strategies were discerned in the topics of leadership, change management, buy-in, engagement, workflow, finance and human resources, training, ethics, legal and data questions, evaluation and monitoring, and maintenance. The study has also identified that more knowledge is needed and future research should focus on identifying leadership’s contribution and actions, contractual arrangements between actors such as hospitals, developers, universities, legal strategies given the uncertainties in the regulatory framework of AI in healthcare, how ethical questions can be resolved, what kind of infrastructure is necessary for a hospital to be able to effectively implement AI, and what are the decision points for suspending the implementation, removing the AI system from practice and what would the related consequences be. Further research should also aim to synthesize knowledge on barriers and strategies into practical and actionable methodologies that could guide ecosystems in AI implementations. With many AI systems struggling to get integrated into the healthcare system’s routines, guidance for implementation teams is necessary, as it can condition better chances for implementations to succeed.

## Supporting information

S1 Appendix Comparison of concepts. (DOCX)

S2 Appendix Interview guide. (DOCX)
