# Supplementary material for: A comprehensive overview of barriers and strategies for AI implementation in healthcare: Mixed-method design
Source: PLoS One. 2024 Aug 9;19(8):e0305949. doi: 10.1371/journal.pone.0305949 (PMC11315296; doi:10.1371/journal.pone.0305949)
Supplement: S1 Appendix — (DOCX) [file pone.0305949.s001.docx]

|  | Concepts used in the present study | Concepts used in the included scoping and systematic literature reviews |
| --- | --- | --- |
| 1 | Leadership | - Sharma et al. [18]: None - Chomutare et al. [19]: Leadership - Chan et al. [20]: None - Tricco et al. [21]: Requires leadership support - van der Vegt et al. [22]: None - Lee et al. [23]: None |
| 2 | Change management | - Sharma et al. [18]: Communication, Champions, Incentives - Chomutare et al. [19]: Communication, Organizational policy and culture, Champions - Chan et al. [20]: Tool/model advocated by a professional or regulatory body - Tricco et al. [21]: None - van der Vegt et al. [22]: Communication, Clinical champions - Lee et al. [23]: Champions, Incentives and reminders |
| 3 | Buy-in | - Sharma et al. [18]: Communication - Chomutare et al. [19]: Trialability, Motivation, Interpretability, Usability, Experiences and prior knowledge, Trust, Prior evidence, Interpretability, Complexity - Chan et al. [20]: Clinical utility, Model performance, Trust, Model suitability for disease in a context, Model complementarity with specific experience in existing staff - Tricco et al. [21]: Reliability of technology, Customization, Transparency, Diagnostic accuracy, Perceived usefulness, Over-reliance on Machine Learning, Attitude toward Machine Learning, Visual readability, Easiness of use, Timeliness of output, Targeting of specific goals - van der Vegt et al. [22]: Trust, Alert fatigue, Perceptions of role and added value, Concerns about overreliance on AI system - Lee et al. [23]: Buy-in, Alert fatigue, Technological limitations of AI system, Added value of AI system |
| 4 | Engagement | - Sharma et al. [18]: Co-creation, Contextualization, Organizational efforts - Chomutare et al. [19]: Involvement, Patient needs, External collaboration - Chan et al. [20]: Adapting and tailoring to context, Developing stakeholder relationships - Tricco et al. [21]: Involvement of clinicians into implementation, Communication among clinicians/ care teams - van der Vegt et al. [22]: Clinician involvement - Lee et al. [23]: Fit for institution, Engagement |
| 5 | Workflow | - Sharma et al. [18]: Workflow design - Chomutare et al. [19]: Model interoperability, Integration, Adaptability, Disruptiveness - Chan et al. [20]: Adapting and tailoring to context, Changing infrastructure, Existing systems and processes for screening target condition, Disintegrated documentation of model scores - Tricco et al. [21]: Time consumption, Workload, Reminders as support, Integration into existing workflows, Efficiency of care delivery, Alerts - van der Vegt et al. [22]: None - Lee et al. [23]: Workflow, Extra work |
| 6 | Finance and Human Resources | - Sharma et al. [18]: None - Chomutare et al. [19]: Available resources, Cost - Chan et al. [20]: Availability of suitable staff, Existing infrastructure including IT support, Insufficient resources to deal with the consequences of model implementation - Tricco et al. [21]: Resources, Costs/patient insurance, Cost-effectiveness - van der Vegt et al. [22]: Cost for implementation personnel, infrastructure and ongoing maintenance - Lee et al. [23]: Resources, Staffing |
| 7 | Ethics | - Sharma et al. [18]: None - Chomutare et al. [19]: None - Chan et al. [20]: None - Tricco et al. [21]: None - van der Vegt et al. [22]: None - Lee et al. [23]: None |
| 8 | Legal | - Sharma et al. [18]: None. - Chomutare et al. [19]: Regulation and Law - Chan et al. [20]: None - Tricco et al. [21]: None - van der Vegt et al. [22]: None - Lee et al. [23]: None |
| 9 | Data | - Sharma et al. [18]: None - Chomutare et al. [19]: Data availability, Data quality, Data interoperability - Chan et al. [20]: None - Tricco et al. [21]: None - van der Vegt et al. [22]: Limitations of EHR data, Data mismanagement - Lee et al. [23]: Data access |
| 10 | Training | - Sharma et al. [18]: Learning focus, Training - Chomutare et al. [19]: Education of workforce - Chan et al. [20]: Training and educating stakeholders - Tricco et al. [21]: Education, Training - van der Vegt et al. [22]: Test versions for training, education of staff - Lee et al. [23]: End user education |
| 11 | Evaluation and monitoring | - Sharma et al. [18]: None - Chomutare et al. [19]: Evaluation and testing, Generalizability, Usability, Feedback incorporation, Documentation and presentation of results, Prior evidence - Chan et al. [20]: Using evaluative and iterative strategies, Quality improvement - Tricco et al. [21]: Suggestion and improvement request, Use of audit and feedback as support - van der Vegt et al. [22]: Improvement cycles, evaluation bias, surveillance bias - Lee et al. [23]: Validation |
| 12 | Maintenance | - Sharma et al. [18]: Organizational efforts - Chomutare et al. [19]: None - Chan et al. [20]: None - Tricco et al. [21]: None - van der Vegt et al. [22]: None - Lee et al. [23]: None |
