# Supplementary material for: A comprehensive overview of barriers and strategies for AI implementation in healthcare: Mixed-method design
Source: PLoS One. 2024 Aug 9;19(8):e0305949. doi: 10.1371/journal.pone.0305949 (PMC11315296; doi:10.1371/journal.pone.0305949)
Supplement: S2 Appendix — (DOCX) [file pone.0305949.s002.docx]

### **S2 Appendix. Interview guide**

### What is your experience with implementing new technology in healthcare?

### Do you have any thoughts or experiences of your own about applying AI technology to improve healthcare?

### Based on your perspective and your knowledge of AI technology, what opportunities do you see to apply AI in your context?

### What is the problem/challenge and how does this align with the potential AI implementation?

### What kind of familiarity, knowledge and/or previous involvement do you have in work with introducing AI-based solutions?

### How do you see challenges in introducing AI-based solutions?

### Can you describe some areas in healthcare where AI solutions have particularly good potential to contribute to quality improvement?

### What kind of potential improvements can be expected in these areas? Possible results? Impact on patients/population? Impact on employees? Implementation of the working method of information-driven care with the support of AI?

### How do you see AI-based solutions primarily working in practice?

### How will it affect the work that staff are already doing?

### How will it fit into how care for this patient population is currently organized and delivered? What would need to be changed?

### Which professional groups will primarily be affected? How?

### How do you think AI-based solutions will affect you in your professional role?

### What resources and time do you think it will require of you?

### Do you see potential unexpected and/or unavoidable consequences that could come through AI-based solutions?

### What possible obstacles do you see? What risks do you see?

### How would you generally define success when implementing an AI- based solution?

### What resources and/or time do you think will be required to carry out such implementation? Is there specific time/resources to support this?
